# The Association of Post-Concussion and Post-Traumatic Stress Disorder Symptoms with Health-Related Quality of Life, Health Care Use and Return-to-Work after Mild Traumatic Brain Injury

**DOI:** 10.3390/jcm10112473

**Published:** 2021-06-02

**Authors:** Marjolein van der Vlegel, Suzanne Polinder, Ana Mikolic, Rana Kaplan, Nicole von Steinbuechel, Anne Marie Plass, Marina Zeldovich, Dominique van Praag, Fabian Bockhop, Katrin Cunitz, Isabelle Mueller, Juanita A. Haagsma, The CENTER-TBI Participants and Investigators

**Affiliations:** 1 Department of Public Health, Erasmus MC, 3000 CA Rotterdam, The Netherlands; s.polinder@erasmusmc.nl (S.P.); a.mikolic@erasmusmc.nl (A.M.); z.kaplan@erasmusmc.nl (R.K.); j.haagsma@erasmusmc.nl (J.A.H.); 2 Institute of Medical Psychology and Medical Sociology, University Medical Center Göttingen (UMG), Georg-August-University, 37073 Göttingen, Germany; nvsteinbuechel@med.uni-goettingen.de (N.v.S.); annemarie.plass@med.uni-goettingen.de (A.M.P.); marina.zeldovich@med.uni-goettingen.de (M.Z.); fabian.bockhop@med.uni-goettingen.de (F.B.); katrin.cunitz@med.uni-goettingen.de (K.C.); isabelle.mueller@med.uni-goettingen.de (I.M.); 3 Department of Psychology, Antwerp University Hospital, University of Antwerp, 2650 Edegem, Belgium; Dominique.VanPraag@uza.be

**Keywords:** traumatic brain injury (TBI), post-concussion symptoms, Rivermead Post-Concussion Symptoms Questionnaire (RPQ), post-traumatic stress disorder, health-related quality of life, TBI outcome

## Abstract

Patients with mild traumatic brain injury (mTBI) are at risk for post-concussion (PC) symptoms and post-traumatic stress disorder (PTSD). The co-occurrence of PC and PTSD symptoms after mTBI in relation to health-related quality of life (HRQoL), health care utilization, and return to work has not yet been investigated. PC and PTSD symptoms were measured six months post-TBI by respectively the Rivermead Post-Concussion Symptoms Questionnaire (RPQ) and the Post-Traumatic Stress Disorder Checklist for DSM-5 (PCL-5). Of the 1566 individuals after mTBI who met the inclusion criteria, 26.1% experienced PC symptoms (RPQ ≥16). Additionally, 9.8% experienced PTSD symptoms (PCL-5 ≥ 33), of which the vast majority (81%) also reported experiencing PC symptoms. Differences between patients with no/mild symptoms, with only PC, only PTSD, and both PC and PTSD symptoms in HRQoL, return to work, and rehabilitation were analyzed using logistic and linear regression analyses. Patients with PC and/or PTSD symptoms reported lower HRQoL, higher rates of rehabilitation, and lower return to work rates compared to patients with no/mild symptoms. Patients with both PC and PTSD symptoms reported significantly lower HRQoL (B = −2.73, CI = −4.65; −0.83, *p* < 0.001) compared to those with only PC symptoms, while there were no significant differences in their ongoing rehabilitation care (OR = 1.39, CI = 0.77–2.49, *p* = 0.272) and return to work rates (OR = 0.49, CI = 0.15–1.63, *p* = 0.246) at six months. These results underline the importance of the diagnosis and appropriate treatment of patients with mTBI, experiencing PC and/or PTSD symptoms.

## 1. Introduction

Traumatic brain injury (TBI) is an important public health problem with more than 50 million yearly cases worldwide [1]. It can be defined as ‘an alteration in brain function, or other evidence of brain pathology, caused by an external force’ [2]. The vast majority (70–90%) of patients are classified as having mild TBI (mTBI) [1]. Individuals after mTBI may experience short and long-term physical, psychiatric, emotional, and cognitive disabilities [3,4].

Patients with mTBI can suffer from various post-concussion (PC) symptoms and in some cases, these symptoms can persist for months [5,6,7,8]. PC symptoms can manifest as somatic problems (e.g., fatigue, headache, blurred vision), cognitive deficits (e.g., poor concentration, memory difficulty), or emotional and/or behavioral problems (e.g., depression, frustration, restlessness). Patients may also suffer from symptoms of post-traumatic stress disorder (PTSD) [6,9]. After mild TBI in the civilian setting, approximately 14% have PTSD [10]. These symptoms can be differentiated into four symptom groups: intrusion/re-experiencing, avoidance, negative alterations in cognition and mood, and increased arousal/reactivity. A clinical diagnosis of PTSD requires the presence of symptoms of all four groups [11,12]. Conceptually, there is an overlap between PC and PTSD symptoms due to similarities between PC symptoms and symptoms associated with the hyper- arousal dimension of PTSD (e.g., concentration problems, sleep disturbances) [13,14]. As a result of the overlapping symptoms, some previous studies suggested that PC symptoms should be considered part of the arousal/reactivity subscale of PTSD [13]. However, it is also suggested that there is not only an overlap between post-concussion and PTSD symptoms but a possible interaction [15]. PTSD symptoms could exacerbate PC symptoms, and conversely, PC symptoms could prolong PTSD symptoms.

Experiencing long-term PC and PTSD symptoms after TBI can impair the working ability and the health-related quality of life (HRQoL) of a person [16,17]. Previous literature on patients with mTBI found that 76% of patients reported full return to work six months after injury [18]. Nevertheless, the association between PC and PTSD symptoms with return to work is still unknown. Previous studies have shown that patients with mTBI with PC or PTSD symptoms perceive a lower HRQoL compared to those without these symptoms [16,17,19,20,21]. However, the impact of the co-occurrence of PC and PTSD symptoms on HRQoL, health care utilization, and return to work has to our knowledge not yet been investigated. We hypothesize that patients who report both PC and PTSD symptoms have a lower HRQoL, higher health care utilization, and lower return to work rates. The aim of this study was to investigate the association of PC and PTSD symptoms with HRQoL, health care utilization, and return to work in patients with mTBI.

## 2. Materials and Methods

### 2.1. Study Design and Population

This study was part of the prospective multi-center longitudinal observational Collaborative European NeuroTrauma Effectiveness Research in Traumatic Brain Injury (CENTER-TBI study, registered at ClinicalTrials.gov NCT02210221) [22]. Data were collected from December 2014 to December 2017 in 63 centers in Europe and Israel. Inclusion criteria were a clinical diagnosis of TBI, with an indication for computed tomography (CT) scanning, and presentation to a center within 24 h after injury [22,23]. Patients with a severe pre-existing neurological disorder i.e., cerebrovascular accident, transient ischemic attacks, and epilepsy, which could confound outcome assessments, were excluded. Three strata were used to prospectively differentiate patients by care pathways: emergency room (ER) (discharged after ER visit), admission (primarily admitted to hospital ward), and intensive care unit (ICU) (primarily admitted to ICU). Informed consent was obtained according to local regulations and the Medical Ethics Committees approved the CENTER-TBI study in all participating centers. The main descriptive findings of CENTER-TBI have been previously described [22,23]. In the current study, patients aged 16 years and older, with Glasgow Coma Scale (GCS) 13–15, who completed the Rivermead Post-Concussion Symptoms Questionnaire (RPQ) [24] and the Post-Traumatic Stress Disorder Checklist for the DSM-5 (PCL-5) [25] at six-month follow-up were included.

### 2.2. Measures

#### 2.2.1. Sociodemographic Data

Sex, age, (highest) educational level (primary school, secondary school, post-high school training, college/university), and employment status (full-time employed, part-time employed, unemployed, student, homemaker, retired) were assessed at time of enrollment in the study.

#### 2.2.2. Medical History

Medical history was assessed at time of study enrollment. Pre-injury psychiatric medical history included sleep disorders, depression, anxiety, schizophrenia, drug abuse, or other psychiatric problems as reported by patients.

#### 2.2.3. Injury Characteristics

Overall injury severity was rated by the Injury Severity Score (ISS), which ranges from 0 to 75. It is calculated as the sum of square of the three highest values of the Abbreviated Injury Scale Score (AIS) from different body regions [26]. TBI severity was rated using the Glasgow Coma Scale (GCS) [27]. Participants with a baseline GCS score between 13 and 15 were classified as mild TBI and included in this study.

#### 2.2.4. Functional Outcome at Six Months Post-TBI

Functional outcome was assessed at six months post-TBI using the Glasgow Outcome Scale Extended (GOSE). The GOSE differentiates eight outcome categories: dead (1), vegetative state (2), lower severe disability (3), upper severe disability (4), lower moderate disability (5), upper moderate disability (6), lower good recovery (7), and upper good recovery (8). The categories vegetative state and lower severe disability were combined as responses by postal questionnaire did not permit this differentiation. The GOSE score was dichotomized into incomplete recovery (GOSE < 8) vs. full recovery (GOSE = 8).

#### 2.2.5. Post-Concussion Symptoms at Six Months Post-TBI

Post-concussion symptoms were assessed through the Rivermead Post-Concussion Symptoms Questionnaire (RPQ), which evaluates the frequency and severity of 16 symptoms including headaches, dizziness, nausea/vomiting, noise sensitivity, sleep disturbance, fatigue, being irritable, feeling depressed or tearful, feeling frustrated, or impatient, forgetfulness, poor concentration, taking longer to think, blurred vision, light sensitivity, double vision, and restlessness [24]. For each symptom, patients could respond on a five-point Likert scale (“not experienced at all”, (1) “no more of a problem than before the TBI”, (2) “a mild problem”, (3) “a moderate problem”, and (4) “a severe problem”). For the calculation of the total score, ratings from the 16 items were summed up, excluding the ratings of 1 (“no more of a problem than before”) [24]. The total score ranges from 0 (representing no change in symptoms since TBI) to 64 (most severe symptoms). A total score ≥16 was considered indicative of having severe PC symptoms [28].

#### 2.2.6. Post-Traumatic Stress Symptoms at Six Months Post-TBI

Post-traumatic stress disorder symptoms were assessed with the PCL-5 [25]. The PCL-5 includes 20 items reflecting the DSM-5 diagnostic criteria of PTSD [29]. The items can be divided into four subscales: intrusion (five items), avoidance (two items), negative alterations in cognitions and mood (seven items), and alterations in arousal and reactivity (six items). The self-report rating scale ranges from 0 (not at all) to 4 (extremely) for each symptom and the sum of scores ranges from 0 to 80. A total score ≥33 is considered clinically relevant and was used to screen for PTSD in this study [30]. When referring to patients with PTSD in this study, this classification is solely based on an individual’s PCL-5 score ≥33. Formal diagnosis requires evaluation by a psychiatrist.

#### 2.2.7. Health Care Utilization

Data on hospital and ICU admission, length of hospital stay, length of ICU stay, and inpatient rehabilitation were collected. Both inpatient and outpatient rehabilitation were additionally assessed by a patient-reported questionnaire at 6-months follow-up. Inpatient rehabilitation included admission to a general, TBI specialized, geriatric, psychiatric rehabilitation or nursing home unit. Outpatient rehabilitation included physical therapy, occupational therapy, speech therapy, therapeutic recreation, cognitive remediation, vocational services, psychological services, nursing services, comprehensive day treatment, peer mentoring, social work, independent living, and home health.

#### 2.2.8. Return to Work at Six Months Post-TBI

Return to work was assessed at six-month follow-up. Participants were categorized into three groups: (1) return to work at full level (returned to previous job at same or increased level/hours, change of job), (2) return to work at reduced level/hours (return to previous job at reduced level/hours, sheltered employment), or (3) no return to work (unable to work, looking for work). For return to work, we included employed participants under the age of 65 years in the analyses.

#### 2.2.9. Health Related Quality of Life at Six Months Post-TBI

Generic HRQoL was assessed using the 12-item Short Form Health Survey—Version 2 (SF-12v2) [31]. The HRQoL is summarized as a mental component score (MCS) and a physical component score (PCS), which ranges from 0 to 100. If there was no available SF-12v2 score, the score was derived using the long version of the questionnaire (i.e., the SF-36v2) if available [23]. Scores < 40 are considered to reflect impaired HRQoL [32].

The six-item Quality of Life after Brain Injury Overall Scale (QOLIBRI-OS) is a TBI- specific instrument measuring HRQoL [33]. The instrument assesses the overall satisfaction with life (physical condition, cognition, emotions, function in daily life, personal and social life, and current situation and future prospects). The total score is calculated by computing the mean for the six items and converting this to a percent score by subtracting one and multiplying by 25. The scale can range from 0 to 100, and scores <52 are considered to reflect impaired HRQoL [32].

### 2.3. Ethical Approval

The CENTER-TBI study has been conducted in accordance with all relevant laws of the EU if directly applicable or of direct effect, and all relevant laws of the country where the recruiting sites were located, including, but not limited to, the relevant privacy and data protection laws and regulations (the “Privacy Law”), the relevant laws and regulations on the use of human materials, and all relevant guidance relating to clinical studies from time to time in force including, but not limited to, the ICH Harmonized Tripartite Guideline for Good Clinical Practice (CPMP/ICH/135/95) (“ICH GCP”) and the World Medical Association Declaration of Helsinki entitled “Ethical Principles for Medical Research Involving Human Subjects”. Ethical approval was obtained for each recruiting site. Informed consent was obtained for all patients recruited in the Core Dataset of CENTER-TBI and documented in the e-CRF. The list of sites, ethical committees, approval numbers, and approval dates can be found on the official Center TBI website (www.center-tbi.eu/project/ethical-approval, accessed on 30 April 2021).

### 2.4. Statistical Analysis

Data were extracted from the INCF Neurobot tool version 3.0 (INCF, Solna, Sweden), which is a clinical study data management tool. Descriptive statistics were reported for patient and injury characteristics, and outcome variables. Continuous variables were described with mean and standard deviation (SD) or median and interquartile range (IQR) and categorical data were described with frequencies.

Four groups of patients with and without severe PC and PTSD symptoms were created. Patients were categorized into one of four groups based on RPQ and PCL-5 scores: (1) no/mild symptoms: RPQ < 16 and PCL-5 < 33, (2) PC symptoms: RPQ ≥ 16 and PCL-5 < 33, (3) PC and PTSD symptoms: RPQ ≥ 16 and PCL-5 ≥ 33, (4) PTSD symptoms: RPQ < 16 and PCL-5 ≥ 33. To analyze group differences, the Kruskal–Wallis test for continuous variables and chi-square test for contingency tables for categorical variables were used.

To evaluate the correlation between the RPQ and PCL-5 subscales, Spearman’s correlation coefficients were utilized. A correlation was considered strong when the coefficient was ≥0.5, moderate when the coefficient was between 0.3 and 0.5, and weak when the coefficient was below 0.3 [34].

Multivariate imputation by chained equations was used to impute missing values in potential confounders. All baseline variables, RPQ, PCL-5, SF-12, QoLIBRI-OS, hospital and ICU admission, and rehabilitation care were included in the imputation model. Outcome variables were not imputed. To explore the association between the symptom groups and HRQoL six months post-TBI, multivariable linear regression analyses were performed. Logistic regression analyses were applied to estimate the association between symptom groups and six-month outpatient rehabilitation as well as between symptom groups and return to work at pre-injury level six-months post-TBI. The assumptions of the linear and logistic regression models were met. Additionally, HRQoL of patients with and without ongoing outpatient rehabilitation care and of patients who returned to work versus not returned to work were reported for each symptom group.

For all analyses, a *p*-value of *p* < 0.05 was considered significant. All statistical analyses were performed using SPSS version 25 for Windows (IBM SPSS Statistics, SPSS Inc, Chicago, IL, USA) and R (version 3.5, the R Foundation for Statistical Computing, Vienna, Austria), using the mice package for imputation of missing values.

## 3. Results

### 3.1. Patient Characterstics

**Figure 1:**
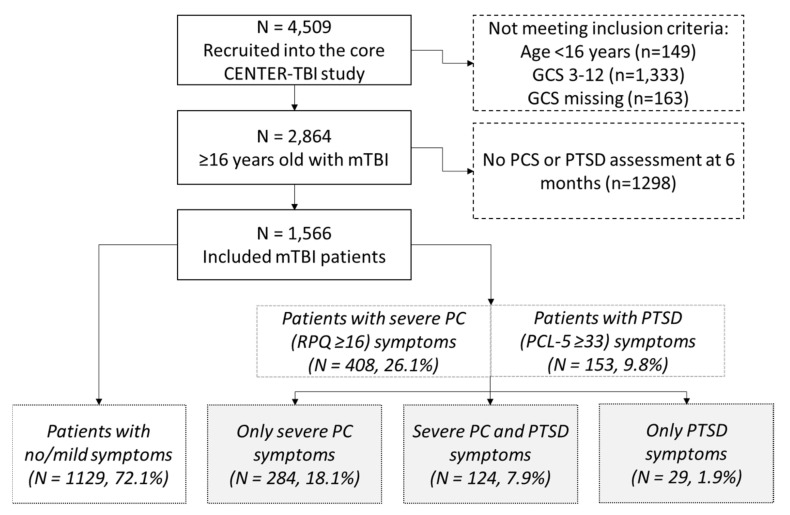
Flow chart. *n* = number; mTBI = mild traumatic brain injury; PCS = post-concussion symptoms; PTSD = post-traumatic stress disorder.

In total, 4509 patients were recruited into the CENTER-TBI study. Of patients ≥ 16 years after mTBI (*n* = 2864), 1566 provided information on PC and PTSD symptoms six months post TBI and were included in this study (Figure 1). As shown in Figure 1, 26.1% (*n* = 408) of the participants reported severe PC symptoms and 9.8% (*n* = 153) of the participants suffered from clinically relevant PTSD symptoms. A total of 7.9% (*n* = 124) of participants met the criteria of both severe PC and clinically relevant PTSD. Baseline characteristics and outcome data are reported in Table 1, which were differentiated for the presence or absence of PC and PTSD symptoms (Table 1). Patients that met the criteria for PTSD were younger and more often had a history of psychiatric problems than those who did not. Patients with severe PC symptoms were more often admitted to the ICU (37.0%) and had the highest ISS (median 13.0) compared to the other groups. At the six-month follow-up, 60.1% of patients with no/mild symptoms had good recovery (GOSE score 8) compared to 13.4% of patients with only severe PC symptoms, 16.1% of patients with severe PC and PTSD symptoms, and 27.6% of patients with only PTSD symptoms (*p* < 0.001).

### 3.2. Prevalence of Post-Concussion and Post-Traumatic Stress Disorder Symptoms

**Figure 2:**
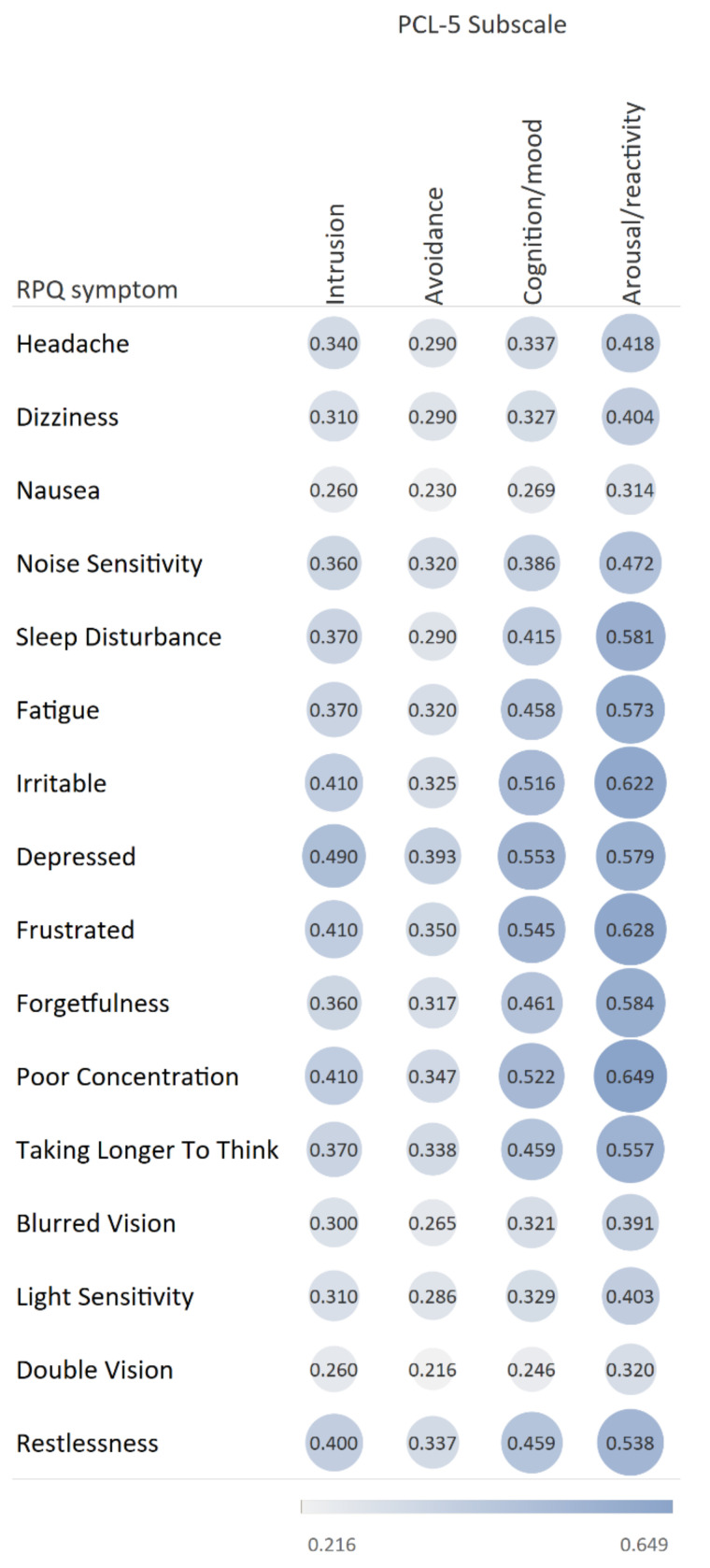
Spearman rank correlations between RPQ symptoms and PCL-5 subscales. All correlations were statistically significant. RPQ: Rivermead Post-Concussion Symptoms Questionnaire; PCL-5: Post-Traumatic Stress Disorder Checklist for the DSM-5.

**Figure A1:**
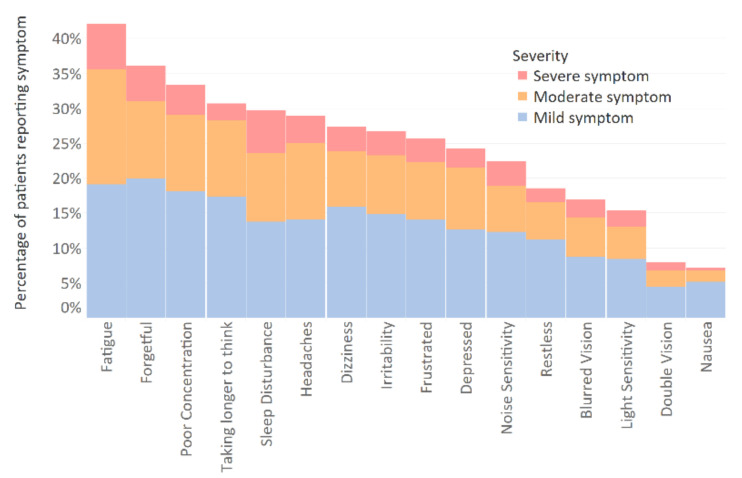
Frequency of post-concussion symptoms as measured by the RPQ 6 months post-TBI.

**Figure A2:**
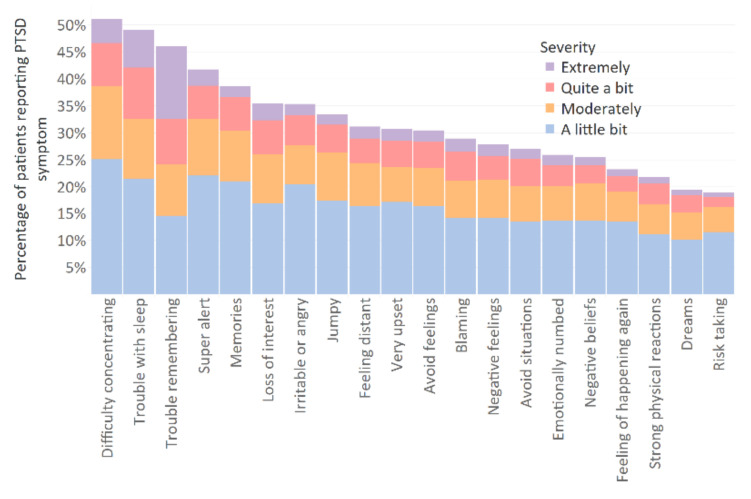
Frequency of post-traumatic stress disorder symptoms as measured by the PCL-5 at 6 months post-TBI.

**Figure A3:**
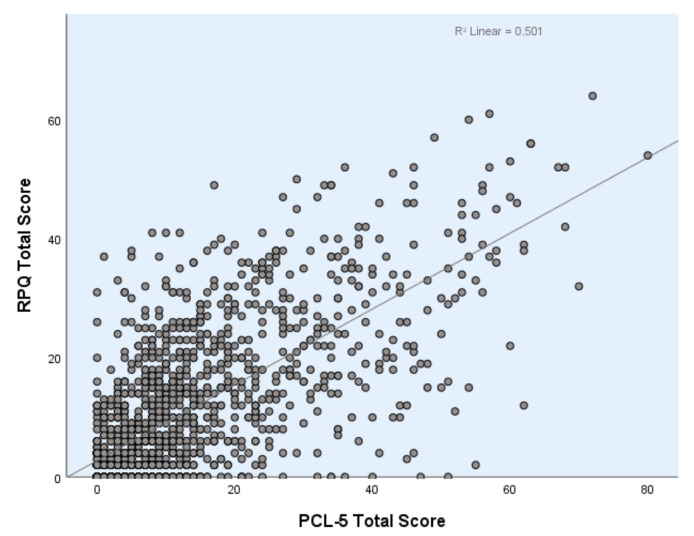
Regression line graph of RPQ total scores and PCL-5 total scores. RPQ: Rivermead Post-Concussion Symptoms Questionnaire; PCL-5: Post-Traumatic Stress Disorder Checklist for the DSM-5.

Six months after TBI, fatigue was the most frequently reported post-concussion symptom (42.1%), followed by ‘forgetfulness’ (36.1%) and ‘poor concentration’ (33.3%) (Figure A1, Appendix A). The most frequently reported PTSD symptoms were ‘difficulty concentrating’ (51.1%), ‘trouble with sleep’ (49.1%), and ‘trouble remembering’ (46.1%) (Figure A2, Appendix A). ‘Light sensitivity’ (15.4%), ‘double vision’ (8.0%), and ‘nausea’ (7.2%) were the least frequently reported post-concussion symptoms and ‘strong physical reactions’ (21.8%), ‘distressing dreams’ (19.4%), and ‘risk taking’ (18.9%) were the least frequently reported PTSD symptoms. Figure 2 shows the Spearman’s correlation coefficients between RPQ symptoms and PCL-5 subscales. A strong correlation was observed between severity of PTSD symptoms in the arousal/reactivity PCL-5 subscale and the majority of RPQ symptoms. The strongest correlations were with ‘poor concentration’ (0.649), ‘frustration’ (0.628), and ‘irritability’ (0.622). Severity of the cognition and mood symptoms PCL-5 subscale had strong correlations with ‘feeling depressed’ (0.553), ‘frustration’ (0.545), ‘poor concentration’ (0.522), and ‘irritability’ (0.516). The correlations between the intrusion and avoidance PCL-5 subscales, and RPQ symptoms were moderate to weak. The total scores of the RPQ showed a positive correlation with the total scores of the PCL-5 (r = 0.706, *p* < 0.001) (Figure A3, Appendix A).

### 3.3. Differences in HRQoL, Health Care Utilization, and Return to Work among the Severe PC and PTSD Groups

Patients with severe PC and/or PTSD symptoms at six months post TBI reported lower HRQoL scores than patients with no/mild symptoms, with lowest scores for patients with both severe PC and PTSD symptoms (Table 2). After adjustment for age, sex, educational level, psychiatric history, and ISS, patients with severe PC and PTSD symptoms had significantly lower HRQoL scores than patients with only severe PC symptoms, based on SF-12 physical component score (B −2.73, 95% CI −4.64; −0.83, *p* = 0.001), SF-12 mental component score (B −9.37, 95% CI −11.28; −7.46, *p* < 0.001), and QoLIBRI-OS (B −11.87, 95% CI −15.49; −8.24, *p* < 0.001) (Table A1, Appendix B).

Patients with severe PC and/or PTSD symptoms at six months post TBI were more likely to have been admitted to a hospital ward or ICU and to receive inpatient and outpatient rehabilitation care after sustaining their TBI compared to patients not meeting criteria for severe PC and PTSD symptoms (Table 3). After adjustment for age, sex, educational level, psychiatric history, and ISS, there were no significant differences in hospital and ICU admission or inpatient rehabilitation between groups, with the exception of a higher likelihood of ICU admission after TBI for patients with severe PC symptoms compared to patients no/mild symptoms (OR 0.54, CI 0.36–0.81, *p* = 0.003) and patients with severe PC and PTSD symptoms (OR 0.42, CI 0.20–0.84, *p* = 0.015) (Table A2, Appendix B). For outpatient rehabilitation, there was no significant difference in the likelihood of receiving ongoing rehabilitation care after six months for patients with severe PC symptoms compared to patients with both severe PC and PTSD symptoms (OR 1.39, CI 0.77–2.49, *p* = 0.272). For patients with severe PC and/or PTSD symptoms, HRQoL was comparable to patients who did and did not receive rehabilitation care at six months (Table A3, Appendix B). However, patients not meeting criteria for severe PC and PTSD symptoms, who received care at six months reported significantly lower HRQoL compared to those who did not. Additionally, those patients with ongoing rehabilitation care at six months had higher ISS in each group compared to those not receiving rehabilitation care after six months (Table A4, Appendix B).

Table 4 shows the proportion of patients that returned to work within six months post-TBI. There were 760 employed participants aged 16–65 years. While 83.2% of patients with no/mild symptoms returned to work at pre-injury level at six months after TBI, patients with severe PC symptoms, PTSD symptoms, or both severe PC and PTSD symptoms had return-to-work rates of respectively 46.1%, 62.5%, and 59.0%. After adjustment for age, sex, educational level, psychiatric history, and ISS, return to work rates did not differ significantly between patients with only severe PC symptoms and patients with both PC and PTSD symptoms (OR 0.82, 95% CI: 0.42–1.63, *p* = 0.595) or only PTSD symptoms (OR 0.49, 95% CI: 0.15–1.63, *p* = 0.246) (Table A5, Appendix B). Within each group, SF-12 physical component scores were significantly lower for those who reported no full return to work within six months, while SF-12 mental component scores were comparable for patients with and without return to work. Additionally, patients with severe PC and/or PTSD symptoms who returned to work reported higher SF-12 physical component scores than patients with no/mild symptoms who did not return to work (Table A6, Appendix B). The ISS of patients not returning to work at six months was higher compared to those that did return to work. The ISS of patients with no/mild symptoms who did not return to work within six months was twice as high (mean 22.1, SD 15.0) than for patients who did return to work at full level (mean 11.3, SD 8.8) (*p* < 0.001) (Table A7, Appendix B).

## 4. Discussion

This study aimed to investigate the co-occurrence of PC and PTSD symptoms in patients with mTBI and the association of these symptoms with HRQoL, health care utilization, and return to work. Six months after mTBI, one in four patients experienced severe PC symptoms. Furthermore, nearly 10% experienced symptoms indicative of PTSD, of which the vast majority (81%) also reported experiencing severe PC symptoms. The correlation between post-concussion symptoms and symptoms of the PTSD arousal/reactivity subscale was strong. Our study showed that severe PC and/or PTSD symptoms were associated with lower HRQoL, higher use of rehabilitation care, and lower return to work rates. We found that patients experiencing both PC and PTSD symptoms reported the lowest HRQoL, while use of rehabilitation care and return to work rates were comparable between patients with only PC symptoms and both severe PC and PTSD symptoms.

The correlation found between PC symptoms and symptoms of the PTSD arousal/reactivity subscale is in accordance with a previous study that also showed this correlation in mild TBI patients [13]. As a result of the high correlation between several PC and PTSD symptoms, in our study, most patients with probable PTSD also score higher on PC symptoms. Reversely, most patients with severe PC symptoms do not meet the criteria for PTSD apart from those with very high RPQ scores. The overlapping symptoms complicate accurate attribution of the cause of these symptoms to a specific disorder. This has important implications for the treatment of the patient. Diagnosis of PTSD in persons six months after TBI might disguise the proper diagnosis of PC symptoms since the high PTSD scores might be due to the high PC symptom burden these people still experience. As a result of the large overlap in PC or PTSD symptoms and their co-occurrence in relationship with other outcomes, including HRQoL, long-term rehabilitation, and return to work, caution is warranted in linking disorders independently to adverse outcomes.

A previous systematic review on HRQoL in patients who sustained a TBI showed that in the long-term, patients still showed large deviations from full recovery when measured by population norms [35]. Our study showed substantial differences between patients with mTBI, with and without severe PC, and PTSD symptoms. Patients with no/mild symptoms had HRQoL scores comparable to population norms from several European countries, indicating that this group had good recovery [36,37]. Previous studies have reported on the association between PC symptoms or PTSD with lower HRQoL [16,17]. An important finding of our study is that severe PC and PTSD symptoms possibly intensify each other, since patients with combined symptoms had the lowest HRQoL. Another explanation for these low HRQoL scores could be the high severity of PC symptoms in the group with both severe PC and PTSD symptoms.

Our study showed that in total, 74.3% of patients with mTBI return to work at pre-injury level, which is in accordance with previous literature [18,38]. However, our results did indicate significant differences between patients with and without PC and PTSD symptoms. Around half of all participants with severe PC and/or PTSD symptoms have not resumed work at a pre-injury level. These symptoms might affect physical, psychosocial, and cognitive skills, and therefore one’s ability to work [39,40]. While patients with mTBI with no or mild symptoms after six months are expected to show good recovery, a subgroup of these patients still received rehabilitation care six months post TBI and did not return to work at that time. However, there may be other causes that prevent patients from full recovery. One explanation for this could be the presence of extra-cranial injury, since these patients showed physical HRQoL scores that were below population norms. Another explanation might be the higher ISS scores indicating multiple injuries, due to which the rehabilitation process lasts longer. Additionally, patients with severe PC symptoms had higher ISS than those without these symptoms, which might explain the lower HRQoL scores for this group. However, the presence of PC likely complicates recovery not just for organic reasons but for secondary psychological effects [41]. While early PC may be best described as a neurological problem, the chronic presence of PC likely includes the development of neuropsychiatric symptoms [42]. The typical symptoms of PC are difficult to treat and may encourage maladaptive coping methods, further complicating recovery. Moreover, the often associated loss of employment, social role, and overall HRQoL can be psychologically challenging and may increase the likelihood of emerging dysphoria, dysthymia, or depression, especially in patients with pre-existing risk factors for mood disorders [43]. The rise of these secondary psychiatric symptoms may further complicate a successful treatment of PC [44]. This underlines the complexity of the recovery after TBI.

### 4.1. Strengths and Limitations

In this study, a large dataset was used including patients from hospital centers across Europe. Previous studies focused exclusively on either post-concussion or PTSD symptoms in relation to other outcomes. Our study underlines the importance of measuring both, since HRQoL was lowest for patients experiencing both. That could suggest a possible interaction and mutual exacerbation of symptoms. However, the severity of PC symptoms in this group could be another explanation for these results.

There were some limitations to this study. First, patients were categorized in one of four groups based on self-reported RPQ and PCL-5 data. However, the use of these questionnaires is not sufficient to clinically diagnose patients with, for example, PTSD, where the gold standard is a (semi-)structured interview. Additionally, it is important to note that there is not one optimal RPQ and PCL-5 cut-off, as previous studies recommended a variety of cut-off scores [28,30,45,46]. This lack of standardized cut-off points may have affected the magnitude of the odds ratio (OR) as well [47]. While OR is widely used as an indicator of risk for disease, it can vary strongly depending on sample size, case rates, and applied cut-off scores in data [48]. Furthermore, CENTER-TBI participants (see Supplementary Materials) were mainly recruited from trauma referral centers. This may be a selected sample of neurotrauma centers, limiting generalizability to all European TBI patients.

### 4.2. Outlook

Identification of patients with PC and/or PTSD symptoms and medical and psychological interventions for those specific patients might be effective in prevention of long-term post-concussion and PTSD symptoms. In turn, the need for long-term rehabilitation care might decrease and HRQoL and return-to-work rates might increase [49,50]. This is important, since half of the patients with symptoms did not return to their previous work level, causing a large burden for both the patient and society. Clinicians should not overlook a diagnosis of post-concussion when a patient also reports PTSD symptoms. Additionally, future prospective studies could clarify the possible causal relationship and interaction between PC and PTSD symptoms and their relationship with other outcomes. We conclude that there is a need for paying attention to the diagnosis of patients with mTBI, experiencing post-concussion and/or PTSD symptoms, to ensure appropriate interventions and to facilitate recovery.

## Figures and Tables

**Table 1:** Descriptive statistics of the total sample and for patients with severe PC and/or PTSD symptoms, at six months post-mTBI.

Variable	Total Population	No/Mild Symptoms ^1^	Severe PC Symptoms, no PTSD ^2^	Severe PC and PTSD Symptoms ^3^	PTSD, no Severe PC Symptoms ^4^	*p*-Value
*N*	1566	1129	284	124	29	
Age, median (IQR)	53.0 (35.0–66.0)	53.0 (34.0–67.0)	56.0 (41.5–66.8)	49.0 (32.0–59.8)	47.0 (30.5–59.5)	0.001
Sex, male	993 (63.4%)	752 (66.6%)	145 (51.1%)	77 (62.1%)	19 (65.5%)	<0.001
Highest level of education						0.017
Primary	202 (12.9%)	138 (12.2%)	39 (13.7%)	15 (12.1%)	10 (34.5%)	
Secondary	452 (28.9%)	324 (28.7%)	81 (28.5%)	41 (33.1%)	6 (20.7%)	
Post-high school training	291 (18.6%)	199 (17.6%)	60 (21.1%)	28 (22.6%)	4 (13.8%)	
College/University	473 (30.2%)	363 (32.2%)	73 (25.7%)	30 (24.2%)	7 (24.1%)	
NA	148 (9.5%)	105 (9.3%)	31 (10.9%)	10 (8.1%)	2 (6.9%)	
Baseline employment						<0.001
Full-time employed	626 (40.0%)	460 (40.7%)	108 (38.0%)	51 (41.1%)	7 (24.1%)	
Part-time employed	195 (12.5%)	124 (11.0%)	42 (14.8%)	20 (16.1%)	9 (31.0%)	
Unemployed	91 (5.8%)	51 (4.5%)	21 (7.4%)	16 (12.9%)	3 (10.3%)	
Student	139 (8.9%)	108 (9.6%)	19 (6.7%)	9 (7.3%)	3 (10.3%)	
Homemaker	22 (1.4%)	8 (0.7%)	10 (3.5%)	2 (1.6%)	2 (6.9%)	
Retired	406 (25.9%)	315 (27.9%)	70 (24.6%)	17 (13.7%)	4 (13.8%)	
NA	87 (5.6%)	63 (5.6%)	14 (4.9%)	9 (7.3%)	1 (3.4%)	
Care pathway						<0.001
ER	555 (28.4%)	346 (30.6%)	58 (20.4%)	33 (26.6%)	7 (24.1%)	
Hospital ward	752 (48.0%)	556 (49.2%)	121 (42.6%)	61 (49.2%)	14 (48.3%)	
ICU	370 (23.6%)	227 (20.1%)	105 (37.0%)	30 (24.2%)	8 (27.6%)	
Pre-injury psychiatric condition						<0.001
Yes	190 (12.1%)	100 (8.9%)	49 (17.3%)	32 (25.8%)	9 (31.0%)	
No	1369 (87.4%)	1025 (90.8%)	233 (82.0%)	91 (73.4%)	20 (69.0%)	
NA	7 (0.4%)	4 (0.4%)	2 (0.7%)	1 (0.8%)	0 (0.0%)	
ISS, median (IQR)	10.0 (5.0–18.0)	9.0 (5.0–17.0)	13.0 (9.0–25.0)	9.0 (4.0–18.0)	11.0 (7.0–17.5)	<0.001
6-month outcomes						
Full recovery (GOSE = 8)	745 (47.6%)	679 (60.1%)	38 (13.4%)	20 (16.1%)	8 (27.6%)	<0.001
RPQ total score, median (IQR)	6.0 (0.0–16.0)	2.0 (0.0–7.0)	25.0 (19.0–29.8)	34.0 (24.0–42.0)	8.0 (2.5–13.0)	<0.001
PCL-5 total score, median (IQR)	7.0 (2.0–16.0)	4.0 (1.0–9.0)	16.3 (10.0–23.0)	45.5 (37.0–53.0)	42.5 (35.0–47.5)	<0.001

^1^ No/mild symptoms: RPQ < 16 and PCL-5 < 33. ^2^ Severe PC symptoms, no PTSD: RPQ ≥ 16 and PCL-5 < 33. ^3^ Severe PC and PTSD symptoms: RPQ ≥ 16 and PCL-5 ≥ 33. ^4^ PTSD, no severe PC symptoms: RPQ < 16 and PCL-5 ≥ 33. Abbreviations: PC = post-concussion, PTSD = post-traumatic stress disorder, mTBI = mild traumatic brain injury, SD = standard deviation, IQR = interquartile range, GCS = Glasgow Coma Scale, ISS = Injury Severity Score, NA = not available.

**Table 2:** Health-related quality of life by PC and PTSD symptom status, 6 months post-TBI.

HRQoL Measure	No/Mild Symptoms ^1^	Severe PC Symptoms, No PTSD ^2^	Severe PC and PTSD Symptoms ^3^	PTSD, No Severe PC Symptoms ^4^
Participants	*n* = 1129	*n* = 284	*n* = 124	*n* = 29
SF-12 PCS	48.9 * (9.3)	40.2 (11.1)	39.2 * (10.7)	47.3 * (10.6)
SF-12 MCS	52.0 * (8.7)	41.3 (10.2)	31.6 * (9.6)	36.9 * (8.9)
QoLIBRI-OS	77.1 * (16.6)	54.5 (18.6)	42.3 * (21.5)	52.6 (21.3)

* Linear regression analysis on HRQoL at 6 months. Statistically significant (*p* < 0.05) difference compared to the ‘only PC group’ after adjusted for age, sex, educational level, psychiatric history, and injury severity score. ^1^ No/mild symptoms: RPQ < 16 and PCL-5 < 33. ^2^ Severe PC symptoms, no PTSD: RPQ ≥ 16 and PCL-5 < 33. ^3^ Severe PC and PTSD symptoms: RPQ ≥ 16 and PCL-5 ≥ 33. ^4^ PTSD, no severe PC symptoms: RPQ < 16 and PCL-5 ≥ 33. Abbreviations: PC = post-concussion, PTSD = post-traumatic stress disorder, HRQoL = health-related quality of life, SF-12 PCS = 12-item Short Form Health Survey—Version 2 physical component score, SF-12 PCS = 12-item Short Form Health Survey—Version 2 mental component score, QoLIBRI-OS = Quality of Life after Brain Injury Overall Scale.

**Table 3:** Health care utilization by PC and PTSD symptom status.

Health Care Utilization	No/Mild Symptoms ^1^	Severe PC Symptoms, No PTSD ^2^	Severe PC and PTSD Symptoms ^3^	PTSD, No Severe PC Symptoms ^4^
Participants	*n* = 1129	*n* = 284	*n* = 124	*n* = 29
In-hospital				
Admission at hospital ward	67.6%	74.8%	67.7%	79.3%
*Hospital length of stay in days* ^5^	*5.7 (10.6)*	*6.8 (9.1)*	*8.6 (11.5)*	*7.3 (8.6)*
Admission at ICU	21.0% *	37.9%	24.2% *	27.6%
*ICU length of stay in days* ^5^	*5.0 (7.4)*	*4.9 (7.6)*	*8.7 (10.9)*	*5.6 (6.4)*
Inpatient rehabilitation	10.9%	17.4%	13.7%	17.2%
Outpatient rehabilitation	15.7% *	31.2%	30.9%	24.1%
*Ongoing after six months* ^6^	*6.5%*	*18.4%*	*19.5%*	*6.9%*

* Logistic regression analysis on health care utilization. Statistically significant (*p* < 0.05) difference compared to the ‘only PC group’ after adjusted for age, sex, educational level, psychiatric history, and Injury Severity Score. ^1^ No/mild symptoms: RPQ < 16 and PCL-5 < 33. ^2^ Severe PC symptoms, no PTSD: RPQ ≥ 16 and PCL-5 < 33. ^3^ Severe PC and PTSD symptoms: RPQ ≥ 16 and PCL-5 ≥ 33. ^4^ PTSD, no severe PC symptoms: RPQ < 16 and PCL-5 ≥ 33. ^5^ Mean (SD) number of days for those admitted. ^6^ Percentage of patients that received ongoing outpatient rehabilitation six months after TBI. Abbreviations: PC = post concussion, PTSD = post-traumatic stress disorder, ICU = intensive care unit.

**Table 4:** Return to work at 6 months post TBI by PC and PTSD symptoms status.

Return to Work	Total Population	No/Mild Symptoms ^1^	Severe PC Symptoms, No PTSD ^2^	Severe PC and PTSD Symptoms ^3^	PTSD, No Severe PC Symptoms ^4^
Participants	*n* = 1566	*n* = 1129	*n* = 284	*n* = 124	*n* = 29
Pre-TBI employed participants ^5^	*n* = 760 (48.5%)	*n* = 540 (47.8%)	*n* = 138 (48.6%)	*n* = 66 (53.2%)	*n* = 16 (55.2%)
Return to work at full level	74.3%	83.2% *	46.1%	59.0%	62.5%
Return to work at reduced level/hours	9.5%	8.0%	17.2%	8.2%	0.0%
No return to work	16.2%	8.8%	36.7%	32.8%	37.5%

* Logistic regression analysis on 6-month return to work at full level. Statistically significant (*p* < 0.05) difference compared to the ‘only PC group’ after adjusted for age, sex, educational level, psychiatric history, and Injury Severity Score. ^1^ No/mild symptoms: RPQ < 16 and PCL-5 < 33. ^2^ Severe PC symptoms, no PTSD: RPQ ≥ 16 and PCL-5 < 33. ^3^ Severe PC and PTSD symptoms: RPQ ≥ 16 and PCL-5 ≥ 33. ^4^ PTSD, no severe PC symptoms: RPQ < 16 and PCL-5 ≥ 33. ^5^ 20 missing values (2.6%). Abbreviations: PC = post-concussion, PTSD = post-traumatic stress disorder.

## Data Availability

The data-sharing policy of CENTER-TBI can be found here: https://www.center-tbi.eu/data/sharing (accessed on 30 April 2021).

## Supplementary Materials

The following are available online at https://www.mdpi.com/article/10.3390/jcm10112473/s1, The list of CENTER-TBI participants and investigators.

## Appendix B

**Table A1:** Linear regression analysis on HRQoL at 6 months.

	SF-12 PCS		SF-12 MCS		QOLIBRI-OS Total Score	
	B (95%CI)	*p*-Value	B (95%CI)	*p*-Value	B (95%CI)	*p*-Value
Unadjusted						
No/mild symptoms (ref PC symptoms)	8.77 (7.49; 10.05)	<0.001	10.77 (9.59; 11.96)	<0.001	22.28 (20.01; 24.54)	<0.001
Severe PC and PTSD symptoms (ref PC symptoms)	−0.94 (−3.03; 1.14)	0.374	−9.64 (−11.56; −7.71)	<0.001	−11.13 (−14.83; −7.42)	<0.001
PTSD symptoms (ref PC symptoms)	7.12 (3.38; 10.86)	<0.001	−4.37 (−7.83; −0.91)	0.013	−2.33 (−8.90; 4.24)	0.487
Adjusted ^1^						
No/mild symptoms (ref PC symptoms)	7.24 (6.04; 8.43)	0.005	10.03 (8.83; 11.23)	<0.001	20.58 (18.31; 22.84)	<0.001
Severe PC and PTSD symptoms (ref PC symptoms)	−2.73 (−4.64; −0.83)	0.001	−9.37 (−11.28; −7.46)	<0.001	−11.87 (−15.49; −8.24)	<0.001
PTSD symptoms (ref PC symptoms)	5.99 (2.57; 9.41)	<0.001	−3.66 (−7.10; −0.22)	0.037	−1.60 (−8.02; −4.82)	0.625

^1^ Adjusted for age, sex, educational level, psychiatric history and injury severity score. Abbreviations: PC = post-concussion, PTSD = post-traumatic stress disorder, HRQoL = health-related quality of life, SF-12 PCS = 12-item Short Form Health Survey—Version 2 physical component score, SF-12 PCS = 12-item Short Form Health Survey—Version 2 mental component score, QoLIBRI-OS = Quality of Life after Brain Injury Overall Scale.

**Table A2:** Logistic regression analysis on health care utilization.

	Hospital Ward Admission			ICU Admission			Inpatient Rehabilitation			Ongoing Outpatient Rehabilitation		
	OR	CI (95%)	*p*-Value	OR	CI (95%)	*p*-Value	OR	CI (95%)	*p*-Value	OR	CI (95%)	*p*-Value
Unadjusted												
No/mild symptoms (ref PC symptoms)	0.70	0.52–0.94	0.019	0.44	0.33–0.58	<0.001	0.58	0.41–0.84	0.003	0.31	0.21–0.45	<0.001
Severe PC and PTSD symptoms (ref PC symptoms)	0.71	0.45–1.12	0.141	0.52	0.32–0.84	0.007	0.76	−0.42–1.37	0.358	1.07	0.63–1.84	0.799
PTSD symptoms (ref PC symptoms)	1.29	0.51–3.30	0.595	0.62	0.27–1.46	0.275	0.99	0.36–2.72	0.985	0.33	0.08–1.42	0.136
Adjusted ^1^												
No/mild symptoms (ref PC symptoms)	1.00	0.70–1.41	0.986	0.54	0.36–0.81	0.003	0.95	0.62–1.45	0.818	0.39	0.26–0.60	<0.001
Severe PC and PTSD symptoms (ref PC symptoms)	0.95	0.55–1.63	0.843	0.42	0.20–0.84	0.015	1.25	0.62–2.52	0.538	1.39	0.77–2.49	0.272
PTSD symptoms (ref PC symptoms)	2.04	0.71–5.80	0.184	0.60	0.17–2.12	0.428	1.97	0.61–6.41	0.259	0.37	0.07–1.88	0.231

Abbreviations: PC = post-concussion, PTSD = post-traumatic stress disorder, ICU = intensive care unit, OR = odds ratio. ^1^ Adjusted for age, sex, educational level, psychiatric history, and injury severity score.

**Table A3:** HRQoL for patients who had ongoing rehabilitation care or not at 6 months.

	No/Mild Symptoms		Severe PC Symptoms, No PTSD		PTSD, No Severe PC Symptoms		Severe PC and PTSD Symptoms	
Rehabilitation at 6 Months	No Rehab Care	Ongoing	No Rehab Care	Ongoing	No Rehab Care	Ongoing	No Rehab Care	Ongoing
SF-12 PCS	49.3 (9.1)	43.3 (10.6)	40.6 (11.4)	37.8 (9.8)	47.9 (10.5)	39.2 (11.4)	39.9 (10.5)	36.0 (11.4)
SF-12 MCS	52.2 (8.7)	50.1 (8.5)	41.6 (10.4)	40.4 (8.9)	36.5 (8.6)	42.8 (14.7)	31.3 (9.6)	32.7 (9.9)
QoLIBRI-OS	77.4 (16.6)	71.8 (15.1)	55.4 (18.9)	50.9 (17.0)	52.7 (21.4)	50.0 (29.7)	41.3 (20.5)	45.0 (24.9)

Abbreviations: PC = post-concussion, PTSD = post-traumatic stress disorder, HRQoL = health-related quality of life, SF-12 PCS = 12-item Short Form Health Survey—Version 2 physical component score, SF-12 PCS = 12-item Short Form Health Survey—Version 2 mental component score, QoLIBRI-OS = Quality of Life after Brain Injury Overall Scale.

**Table A4:** Injury Severity Score for patients with and without ongoing rehabilitation at 6 months.

	No/Mild Symptoms		Severe PC Symptoms, No PTSD		PTSD, No Severe PC Symptoms		Severe PC and PTSD Symptoms	
N	540 (47.8%)		138 (48.6%)		16 (55.2%)		66 (53.2%)	
Ongoing rehabilitation at 6 months	No	Yes	No	Yes	No	Yes	No	Yes
ISS, mean (SD)	12.2	20.3	15.0	27.1	13.2	35.0	13.5	18.2
	(9.6)	(12.0)	(11.5)	(17.1)	(10.8)	(33.9)	(12.4)	(13.5)

Abbreviations: PC = post-concussion, PTSD = post-traumatic stress disorder, ISS = Injury Severity Score.

**Table A5:** Logistic regression analysis on 6-month return to work.

	Unadjusted			Adjusted ^1^		
	OR	CI (95%)	*p*-Value	OR	CI (95%)	*p*-Value
No/mild symptoms (ref PC symptoms)	0.17	0.11–0.26	<0.001	0.21	0.14–0.34	<0.001
Severe PC and PTSD symptoms (ref PC symptoms)	0.51	0.18–1.50	0.223	0.49	0.15–1.63	0.246
PTSD symptoms (ref PC symptoms)	0.60	0.32–1.10	0.099	0.82	0.42–1.63	0.575

Abbreviations: PC = post-concussion, PTSD = post-traumatic stress disorder, OR = odds ratio; odds of not returning to work/returning to work at reduced level in relation to odds of returning to work at full level. ^1^ Adjusted for age, sex, educational level, psychiatric history, and injury severity score.

**Table A6:** HRQoL for patients who returned to work at full level or did not at 6 months.

	No/Mild Symptoms		Severe PC Symptoms, no PTSD		PTSD, No Severe PC Symptoms		Severe PC and PTSD Symptoms	
	540 (47.8%)		138 (48.6%)		16 (55.2%)		>66 (53.2%)	
Return to work	Full RTW	No RTW	Full RTW	No RTW	Full RTW	No RTW	Full RTW	No RTW
SF-12 PCS	52.8 (6.3)	43.1 (11.0)	48.2 (7.9)	38.6 (9.2)	54.6 (4.3)	38.9 (8.4)	43.3 (10.8)	36.3 (11.4)
SF-12 MCS	52.7 (7.7)	51.0 (10.0)	41.0 (10.4)	41.7 (9.5)	37.2 (36.6)	34.9 (7.2)	31.9 (9.7)	32.3 (9.1)
QoLIBRI-OS	82.1 (14.3)	70.5 (16.4)	63.4 (17.5)	50.7 (18.2)	58.3 (20.3)	46.5 (20.8)	46.9 (16.4)	45.5 (25.0)

Abbreviations: PC = post-concussion, PTSD = post-traumatic stress disorder, HRQoL = health-related quality of life, SF-12 PCS = 12-item Short Form Health Survey—Version 2 physical component score, SF-12 PCS = 12-item Short Form Health Survey—Version 2 mental component score, QoLIBRI-OS = Quality of Life after Brain Injury Overall Scale, RTW = return to work.

**Table A7:** Injury Severity Score for patients who returned to work at full level or did not at 6 months.

	No Severe PC/PTSD Symptoms		Severe PC Symptoms, No PTSD		PTSD, No Severe PC Symptoms		Severe PC and PTSD Symptoms	
N	540 (47.8%)		138 (48.6%)		16 (55.2%)		66 (53.2%)	
Return to work	Full RTW	No RTW	Full RTW	No RTW	Full RTW	No RTW	Full RTW	No RTW
ISS, mean (SD)	11.3	22.1	13.8	23.7	19.5	14.5	11.3	15.7
	(8.8)	(15.0)	(9.4)	(17.0)	(19.0)	(8.7)	(10.0)	(13.6)

Abbreviations: PC = post-concussion, PTSD = post-traumatic stress disorder, ISS = Injury Severity Score, RTW = return to work.
