# Supplementary material for: The Association of Post-Concussion and Post-Traumatic Stress Disorder Symptoms with Health-Related Quality of Life, Health Care Use and Return-to-Work after Mild Traumatic Brain Injury
Source: J Clin Med. 2021 Jun 2;10(11):2473. doi: 10.3390/jcm10112473 (PMC8199686; doi:10.3390/jcm10112473)
Supplement: Supplementary file 1 [file jcm-10-02473-s001.zip › jcm-1224362-supplementary.pdf]

The CENTER-TBI participants and investigators:

Cecilia Åkerlund[1], Krisztina Amrein[2], Nada Andelic[3], Lasse Andreassen[4], Audny Anke[5], Anna Antoni[6], Gérard Audibert[7], Philippe Azouvi[8], Maria Luisa Azzolini[9], Ronald Bartels[10], Pál Barzó[11], Romuald Beauvais[12], Ronny Beer[13], Bo-Michael Bellander[14], Antonio Belli[15], Habib Benali[16], Maurizio Berardino[17], Luigi Beretta[9], Morten Blaabjerg[18], Peter Bragge[19], Alexandra Brazinova[20], Vibeke Brinck[21], Joanne Brooker[22], Camilla Brorsson[23], Andras Buki[24], Monika Bullinger[25], Manuel Cabeleira[26], Alessio Caccioppola[27], Emiliana Calappi [27], Maria Rosa Calvi[9], Peter Cameron[28], Guillermo Carbayo Lozano[29], Marco Carbonara[27], Simona Cavallo[17], Giorgio Chevallard[30], Arturo Chieregato[30], Giuseppe Citerio[31, 32], Hans Clusmann[33], Mark Coburn[34], Jonathan Coles[35], Jamie D. Cooper[36], Marta Correia[37], Amra Čović [38], Nicola Curry[39], Endre Czeiter[24], Marek Czosnyka[26], Claire Dahyot-Fizelier[40], Paul Dark[41], Helen Dawes[42], Véronique De Keyser[43], Vincent Degos[16], Francesco Della Corte[44], Hugo den Boogert[10], Bart Depreitere[45], Đula Đilvesi [46], Abhishek Dixit[47], Emma Donoghue[22], Jens Dreier[48], Guy-Loup Dulière[49], Ari Ercole[47], Patrick Esser[42], Erzsébet Ezer[50], Martin Fabricius[51], Valery L. Feigin[52], Kelly Foks[53], Shirin Frisvold[54], Alex Furmanov[55], Pablo Gagliardo[56], Damien Galanaud[16], Dashiell Gantner[28], Guoyi Gao[57], Pradeep George[58], Alexandre Ghuysen[59], Lelde Giga[60], Ben Glocker[61], Jagoš Golubovic[46], Pedro A. Gomez [62], Johannes Gratz[63], Benjamin Gravesteijn[64], Francesca Grossi[44], Russell L. Gruen[65], Deepak Gupta[66], Juanita A. Haagsma[64], Iain Haitsma[67], Raimund Helbok[13], Eirik Helseth[68], Lindsay Horton [69], Jilske Huijben[64], Peter J. Hutchinson[70], Bram Jacobs[71], Stefan Jankowski[72], Mike Jarrett[21], Ji-yao Jiang[58], Faye Johnson[73], Kelly Jones[52], Mladen Karan[46], Angelos G. Kolias[70], Erwin Kompanje[74], Daniel Kondziella[51], Evgenios Kornaropoulos[47], Lars-Owe Koskinen[75], Noémi Kovács[76], Ana Kowark[77], Alfonso Lagares[62], Linda Lanyon[58], Steven Laureys[78], Fiona Lecky[79, 80], Didier Ledoux[78], Rolf Lefering[81], Valerie Legrand[82], Aurelie Lejeune[83], Leon Levi[84], Roger Lightfoot[85], Hester Lingsma[64], Andrew I.R. Maas[43], Ana M. Castaño-León[62], Marc Maegele[86], Marek Majdan[20], Alex Manara[87], Geoffrey Manley[88], Costanza Martino[89], Hugues Maréchal[49], Julia Mattern[90], Catherine McMahon[91], Béla Melegh[92], David Menon[47], Tomas Menovsky[43], Ana Mikolic[64], Benoit Misset[78], Visakh Muraleedharan[58], Lynnette Murray[28], Ancuta Negru[93], David Nelson[1], Virginia Newcombe[47], Daan Nieboer[64], József Nyirádi[2], Otesile Olubukola[79], Matej Oresic[94], Fabrizio Ortolano[27], Aarno Palotie[95, 96, 97], Paul M. Parizel[98], Jean-François Payen[99], Natascha Perera[12], Vincent Perlbarg[16], Paolo Persona[100], Wilco Peul[101], Anna Piippo-Karjalainen[102], Matti Pirinen[95], Horia Ples[93], Suzanne Polinder[64], Inigo Pomposo[29], Jussi P. Posti [103], Louis Puybasset[104], Andreea Radoi [105], Arminas Ragauskas[106], Rahul Raj[102], Malinka Rambadagalla[107], Jonathan Rhodes[108], Sylvia Richardson[109], Sophie Richter[47], Samuli Ripatti[95], Saulius Rocka[106], Cecilie Roe[110], Olav Roise[111,112], Jonathan Rosand[113], Jeffrey V. Rosenfeld[114], Christina Rosenlund[115], Guy Rosenthal[55], Rolf Rossaint[77], Sandra Rossi[100], Daniel Rueckert[61] Martin Rusnák[116], Juan Sahuquillo[105], Oliver Sakowitz[90, 117], Renan Sanchez-Porras[117], Janos Sandor[118], Nadine Schäfer[81], Silke Schmidt[119], Herbert Schoechl[120], Guus Schoonman[121], Rico Frederik Schou[122], Elisabeth Schwendenwein[6], Charlie Sewalt[64], Toril Skandsen[123, 124], Peter Smielewski[26], Abayomi Sorinola[125], Emmanuel Stamatakis[47], Simon Stanworth[39], Robert Stevens[126], William Stewart[127], Ewout W. Steyerberg[64, 128], Nino Stocchetti[129],

Nina Sundström[130], Riikka Takala[131], Viktória Tamás[125], Tomas Tamosuitis[132], Mark Steven Taylor[20], Braden Te Ao[52], Olli Tenovuo[103], Alice Theadom[52], Matt Thomas[87], Dick Tibboel[133], Marjolein Timmers[74], Christos Tolias[134], Tony Trapani[28], Cristina Maria Tudora[93], Andreas Unterberg[90], Peter Vajkoczy [135], Shirley Vallance[28], Egils Valeinis[60], Zoltán Vámos[50], Mathieu van der Jagt[136], Gregory Van der Steen[43], Joukje van der Naalt[71], Jeroen T.J.M. van Dijck [101], Thomas A. van Essen[101], Wim Van Hecke[137], Caroline van Heugten[138], Dominique Van Praag[139], Thijs Vande Vyvere[137], Roel P. J. van Wijk[101], Alessia Vargiolu[32], Emmanuel Vega[83], Kimberley Velt[64], Jan Verheyden[137], Paul M. Vespa[140], Anne Vik[123, 141], Rimantas Vilcinis[132], Victor Volovici[67], Nicole von Steinbüchel[38], Daphne Voormolen[64], Petar Vulekovic[46], Kevin K.W. Wang[142], Eveline Wiegers[64], Guy Williams[47], Lindsay Wilson[69], Stefan Winzeck[47], Stefan Wolf[143], Zhihui Yang[113], Peter Ylén[144], Alexander Younsi[90], Frederick A. Zeiler[47,145], Veronika Zelinkova[20], Agate Ziverte[60] , Tommaso Zoerle[27]

[1] Department of Physiology and Pharmacology, Section of Perioperative Medicine and Intensive Care, Karolinska Institutet, Stockholm, Sweden
[2] János Szentágothai Research Centre, University of Pécs, Pécs, Hungary
[3] Division of Surgery and Clinical Neuroscience, Department of Physical Medicine and Rehabilitation, Oslo University Hospital and University of Oslo, Oslo, Norway
[4] Department of Neurosurgery, University Hospital Northern Norway, Tromso, Norway
[5] Department of Physical Medicine and Rehabilitation, University Hospital Northern Norway, Tromso, Norway
[6] Trauma Surgery, Medical University Vienna, Vienna, Austria
[7] Department of Anesthesiology & Intensive Care, University Hospital Nancy, Nancy, France
[8] Raymond Poincare hospital, Assistance Publique – Hopitaux de Paris, Paris, France
[9] Department of Anesthesiology & Intensive Care, S Raffaele University Hospital, Milan, Italy
[10] Department of Neurosurgery, Radboud University Medical Center, Nijmegen, The Netherlands
[11] Department of Neurosurgery, University of Szeged, Szeged, Hungary
[12] International Projects Management, ARTTIC, Munchen, Germany
[13] Department of Neurology, Neurological Intensive Care Unit, Medical University of Innsbruck, Innsbruck, Austria
[14] Department of Neurosurgery & Anesthesia & intensive care medicine, Karolinska University Hospital, Stockholm, Sweden
[15] NIHR Surgical Reconstruction and Microbiology Research Centre, Birmingham, UK
[16] Anesthesie-Réanimation, Assistance Publique – Hopitaux de Paris, Paris, France
[17] Department of Anesthesia & ICU, AOU Città della Salute e della Scienza di Torino - Orthopedic and Trauma Center, Torino, Italy
[18] Department of Neurology, Odense University Hospital, Odense, Denmark
[19] BehaviourWorks Australia, Monash Sustainability Institute, Monash University, Victoria, Australia
[20] Department of Public Health, Faculty of Health Sciences and Social Work, Trnava University, Trnava, Slovakia
[21] Quesgen Systems Inc., Burlingame, California, USA
[22] Australian & New Zealand Intensive Care Research Centre, Department of Epidemiology and Preventive Medicine, School of Public Health and Preventive Medicine, Monash University, Melbourne, Australia
[23] Department of Surgery and Perioperative Science, Umeå University, Umeå, Sweden
[24] Department of Neurosurgery, Medical School, University of Pécs, Hungary and Neurotrauma Research Group, János Szentágothai Research Centre, University of Pécs, Hungary
[25] Department of Medical Psychology, Universitätsklinikum Hamburg-Eppendorf, Hamburg, Germany
[26] Brain Physics Lab, Division of Neurosurgery, Dept of Clinical Neurosciences, University of Cambridge, Addenbrooke's Hospital, Cambridge, UK
[27] Neuro ICU, Fondazione IRCCS Cà Granda Ospedale Maggiore Policlinico, Milan, Italy
[28] ANZIC Research Centre, Monash University, Department of Epidemiology and Preventive Medicine, Melbourne, Victoria, Australia
[29] Department of Neurosurgery, Hospital of Cruces, Bilbao, Spain

[30] NeuroIntensive Care, Niguarda Hospital, Milan, Italy
[31] School of Medicine and Surgery, Università Milano Bicocca, Milano, Italy
[32] NeuroIntensive Care, ASST di Monza, Monza, Italy
[33]Department of Neurosurgery, Medical Faculty RWTH Aachen University, Aachen, Germany
[34] Department of Anesthesiology and Intensive Care Medicine, University Hospital Bonn, Bonn, Germany
[35] Department of Anesthesia & Neurointensive Care, Cambridge University Hospital NHS Foundation Trust, Cambridge, UK
[36] School of Public Health & PM, Monash University and The Alfred Hospital, Melbourne, Victoria, Australia
[37] Radiology/MRI department, MRC Cognition and Brain Sciences Unit, Cambridge, UK
[38] Institute of Medical Psychology and Medical Sociology, Universitätsmedizin Göttingen, Göttingen, Germany
[39] Oxford University Hospitals NHS Trust, Oxford, UK
[40] Intensive Care Unit, CHU Poitiers, Potiers, France
[41] University of Manchester NIHR Biomedical Research Centre, Critical Care Directorate, Salford Royal Hospital NHS Foundation Trust, Salford, UK
[42] Movement Science Group, Faculty of Health and Life Sciences, Oxford Brookes University, Oxford, UK
[43] Department of Neurosurgery, Antwerp University Hospital and University of Antwerp, Edegem, Belgium
[44] Department of Anesthesia & Intensive Care, Maggiore Della Carità Hospital, Novara, Italy
[45] Department of Neurosurgery, University Hospitals Leuven, Leuven, Belgium
[46] Department of Neurosurgery, Clinical centre of Vojvodina, Faculty of Medicine, University of Novi Sad, Novi Sad, Serbia
[47] Division of Anaesthesia, University of Cambridge, Addenbrooke's Hospital, Cambridge, UK
[48] Center for Stroke Research Berlin, Charité – Universitätsmedizin Berlin, corporate member of Freie Universität Berlin, Humboldt-Universität zu Berlin, and Berlin Institute of Health, Berlin, Germany
[49] Intensive Care Unit, CHR Citadelle, Liège, Belgium
[50] Department of Anaesthesiology and Intensive Therapy, University of Pécs, Pécs, Hungary
[51] Departments of Neurology, Clinical Neurophysiology and Neuroanesthesiology, Region Hovedstaden Rigshospitalet, Copenhagen, Denmark
[52] National Institute for Stroke and Applied Neurosciences, Faculty of Health and Environmental Studies, Auckland University of Technology, Auckland, New Zealand
[53] Department of Neurology, Erasmus MC, Rotterdam, the Netherlands
[54] Department of Anesthesiology and Intensive care, University Hospital Northern Norway, Tromso, Norway
[55] Department of Neurosurgery, Hadassah-hebrew University Medical center, Jerusalem, Israel
[56] Fundación Instituto Valenciano de Neurorrehabilitación (FIVAN), Valencia, Spain
[57] Department of Neurosurgery, Shanghai Renji hospital, Shanghai Jiaotong University/school of medicine, Shanghai, China
[58] Karolinska Institutet, INCF International Neuroinformatics Coordinating Facility, Stockholm, Sweden

[59] Emergency Department, CHU, Liège, Belgium
[60] Neurosurgery clinic, Pauls Stradins Clinical University Hospital, Riga, Latvia
[61] Department of Computing, Imperial College London, London, UK
[62] Department of Neurosurgery, Hospital Universitario 12 de Octubre, Madrid, Spain
[63] Department of Anesthesia, Critical Care and Pain Medicine, Medical University of Vienna, Austria
[64] Department of Public Health, Erasmus Medical Center-University Medical Center, Rotterdam, The Netherlands
[65] College of Health and Medicine, Australian National University, Canberra, Australia
[66] Department of Neurosurgery, Neurosciences Centre & JPN Apex trauma centre, All India Institute of Medical Sciences, New Delhi-110029, India
[67] Department of Neurosurgery, Erasmus MC, Rotterdam, the Netherlands
[68] Department of Neurosurgery, Oslo University Hospital, Oslo, Norway
[69] Division of Psychology, University of Stirling, Stirling, UK
[70] Division of Neurosurgery, Department of Clinical Neurosciences, Addenbrooke's Hospital & University of Cambridge, Cambridge, UK
[71] Department of Neurology, University of Groningen, University Medical Center Groningen, Groningen, Netherlands
[72] Neurointensive Care , Sheffield Teaching Hospitals NHS Foundation Trust, Sheffield, UK
[73] Salford Royal Hospital NHS Foundation Trust Acute Research Delivery Team, Salford, UK
[74] Department of Intensive Care and Department of Ethics and Philosophy of Medicine, Erasmus Medical Center, Rotterdam, The Netherlands
[75] Department of Clinical Neuroscience, Neurosurgery, Umeå University, Umeå, Sweden
[76] Hungarian Brain Research Program - Grant No. KTIA_13_NAP-A-II/8, University of Pécs, Pécs, Hungary
[77] Department of Anaesthesiology, University Hospital of Aachen, Aachen, Germany
[78] Cyclotron Research Center , University of Liège, Liège, Belgium
[79] Centre for Urgent and Emergency Care Research (CURE), Health Services Research Section, School of Health and Related Research (ScHARR), University of Sheffield, Sheffield, UK
[80] Emergency Department, Salford Royal Hospital, Salford UK
[81] Institute of Research in Operative Medicine (IFOM), Witten/Herdecke University, Cologne, Germany
[82] VP Global Project Management CNS, ICON, Paris, France
[83] Department of Anesthesiology-Intensive Care, Lille University Hospital, Lille, France
[84] Department of Neurosurgery, Rambam Medical Center, Haifa, Israel
[85] Department of Anesthesiology & Intensive Care, University Hospitals Southhampton NHS Trust, Southhampton, UK
[86] Cologne-Merheim Medical Center (CMMC), Department of Traumatology, Orthopedic Surgery and Sportmedicine, Witten/Herdecke University, Cologne, Germany
[87] Intensive Care Unit, Southmead Hospital, Bristol, Bristol, UK
[88] Department of Neurological Surgery, University of California, San Francisco, California, USA
[89] Department of Anesthesia & Intensive Care,M. Bufalini Hospital, Cesena, Italy
[90] Department of Neurosurgery, University Hospital Heidelberg, Heidelberg, Germany
[91] Department of Neurosurgery, The Walton centre NHS Foundation Trust, Liverpool, UK

[92] Department of Medical Genetics, University of Pécs, Pécs, Hungary
[93] Department of Neurosurgery, Emergency County Hospital Timisoara , Timisoara, Romania
[94] School of Medical Sciences, Örebro University, Örebro, Sweden
[95] Institute for Molecular Medicine Finland, University of Helsinki, Helsinki, Finland
[96] Analytic and Translational Genetics Unit, Department of Medicine; Psychiatric & Neurodevelopmental Genetics Unit, Department of Psychiatry; Department of Neurology, Massachusetts General Hospital, Boston, MA, USA
[97] Program in Medical and Population Genetics; The Stanley Center for Psychiatric Research, The Broad Institute of MIT and Harvard, Cambridge, MA, USA
[98] Department of Radiology, University of Antwerp, Edegem, Belgium
[99] Department of Anesthesiology & Intensive Care, University Hospital of Grenoble, Grenoble, France
[100] Department of Anesthesia & Intensive Care, Azienda Ospedaliera Università di Padova, Padova, Italy
[101] Dept. of Neurosurgery, Leiden University Medical Center, Leiden, The Netherlands and Dept. of Neurosurgery, Medical Center Haaglanden, The Hague, The Netherlands
[102] Department of Neurosurgery, Helsinki University Central Hospital
[103] Division of Clinical Neurosciences, Department of Neurosurgery and Turku Brain Injury Centre, Turku University Hospital and University of Turku, Turku, Finland
[104] Department of Anesthesiology and Critical Care, Pitié -Salpêtrière Teaching Hospital, Assistance Publique, Hôpitaux de Paris and University Pierre et Marie Curie, Paris, France
[105] Neurotraumatology and Neurosurgery Research Unit (UNINN), Vall d'Hebron Research Institute, Barcelona, Spain
[106] Department of Neurosurgery, Kaunas University of technology and Vilnius University, Vilnius, Lithuania
[107] Department of Neurosurgery, Rezekne Hospital, Latvia
[108] Department of Anaesthesia, Critical Care & Pain Medicine NHS Lothian & University of Edinburg, Edinburgh, UK
[109] Director, MRC Biostatistics Unit, Cambridge Institute of Public Health, Cambridge, UK
[110] Department of Physical Medicine and Rehabilitation, Oslo University Hospital/University of Oslo, Oslo, Norway
[111] Division of Orthopedics, Oslo University Hospital, Oslo, Norway
[112] Institue of Clinical Medicine, Faculty of Medicine, University of Oslo, Oslo, Norway
[113] Broad Institute, Cambridge MA Harvard Medical School, Boston MA, Massachusetts General Hospital, Boston MA, USA
[114] National Trauma Research Institute, The Alfred Hospital, Monash University, Melbourne, Victoria, Australia
[115] Department of Neurosurgery, Odense University Hospital, Odense, Denmark
[116] International Neurotrauma Research Organisation, Vienna, Austria
[117] Klinik für Neurochirurgie, Klinikum Ludwigsburg, Ludwigsburg, Germany
[118] Division of Biostatistics and Epidemiology, Department of Preventive Medicine, University of Debrecen, Debrecen, Hungary
[119] Department Health and Prevention, University Greifswald, Greifswald, Germany
[120] Department of Anaesthesiology and Intensive Care, AUVA Trauma Hospital, Salzburg, Austria

[121] Department of Neurology, Elisabeth-TweeSteden Ziekenhuis, Tilburg, the Netherlands
[122] Department of Neuroanesthesia and Neurointensive Care, Odense University Hospital, Odense, Denmark
[123] Department of Neuromedicine and Movement Science, Norwegian University of Science and Technology, NTNU, Trondheim, Norway
[124] Department of Physical Medicine and Rehabilitation, St.Olavs Hospital, Trondheim University Hospital, Trondheim, Norway
[125] Department of Neurosurgery, University of Pécs, Pécs, Hungary
[126] Division of Neuroscience Critical Care, John Hopkins University School of Medicine, Baltimore, USA
[127] Department of Neuropathology, Queen Elizabeth University Hospital and University of Glasgow, Glasgow, UK
[128] Dept. of Department of Biomedical Data Sciences, Leiden University Medical Center, Leiden, The Netherlands
[129] Department of Pathophysiology and Transplantation, Milan University, and Neuroscience ICU, Fondazione IRCCS Cà Granda Ospedale Maggiore Policlinico, Milano, Italy
[130] Department of Radiation Sciences, Biomedical Engineering, Umeå University, Umeå, Sweden
[131] Perioperative Services, Intensive Care Medicine and Pain Management, Turku University Hospital and University of Turku, Turku, Finland
[132] Department of Neurosurgery, Kaunas University of Health Sciences, Kaunas, Lithuania
[133] Intensive Care and Department of Pediatric Surgery, Erasmus Medical Center, Sophia Children's Hospital, Rotterdam, The Netherlands
[134] Department of Neurosurgery, Kings college London, London, UK
[135] Neurologie, Neurochirurgie und Psychiatrie, Charité – Universitätsmedizin Berlin, Berlin, Germany
[136] Department of Intensive Care Adults, Erasmus MC– University Medical Center Rotterdam, Rotterdam, the Netherlands
[137] icoMetrix NV, Leuven, Belgium
[138] Movement Science Group, Faculty of Health and Life Sciences, Oxford Brookes University, Oxford, UK
[139] Psychology Department, Antwerp University Hospital, Edegem, Belgium
[140] Director of Neurocritical Care, University of California, Los Angeles, USA
[141] Department of Neurosurgery, St.Olavs Hospital, Trondheim University Hospital, Trondheim, Norway
[142] Department of Emergency Medicine, University of Florida, Gainesville, Florida, USA
[143] Department of Neurosurgery, Charité – Universitätsmedizin Berlin, corporate member of Freie Universität Berlin, Humboldt-Universität zu Berlin, and Berlin Institute of Health, Berlin, Germany
[144] VTT Technical Research Centre, Tampere, Finland
[145] Section of Neurosurgery, Department of Surgery, Rady Faculty of Health Sciences, University of Manitoba, Winnipeg, MB, Canada

Åkerlund	Cecilia	cecilia.ai.akerlund@gmail.com
Amrein	Krisztina	tina.amrein84@gmail.com
Andelic	Nada	NADAND@ous-hf.no
Andreassen	Lasse	Lasse.Andreassen@unn.no
Anke	Audny	Audny.anke@unn.no
Antoni	Anna	anna.antoni@meduniwien.ac.at
Audibert	Gérard	g.audibert@chu-nancy.fr
Azouvi	Philippe	philippe.azouvi@rpc.aphp.fr
Azzolini	Maria Luisa	azzolini.marialuisa@hsr.it
Bartels	Ronald	Ronald.Bartels@radboudumc.nl
Barzó	Pál	pbarzo@gmail.com
Beauvais	Romuald	beauvais@arttic.eu
Beer	Ronny	ronny.beer@i-med.ac.at
Bellander	Bo-Michael	bo-michael.bellander@karolinska.se
Belli	Antonio	a.belli@bham.ac.uk
Benali	Habib	habib.benali@gmail.com
Berardino	Maurizio	maurizio_berardino@fastwebnet.it
Beretta	Luigi	beretta.luigi@hsr.it
Blaabjerg	Morten	morten.blaabjerg1@rsyd.dk
Bragge	Peter	peter.bragge@monash.edu
Brazinova	Alexandra	alexandra.brazinova@gmail.com
Brinck	Vibeke	vibeke.brinck@quesgen.com
Brooker	Joanne	Joanne.Brooker@monash.edu
Brorsson	Camilla	Camilla.Brorsson@umu.se
Buki	Andras	2saturn@gmail.com
Bullinger	Monika	bullinger@uke.de
Cabeleira	Manuel	mc916@cam.ac.uk
Caccioppola	Alessio	alessio.caccioppola@gmail.com
Calappi	Emiliana	calemy02@yahoo.it
Calvi	Maria Rosa	calvi.mariarosa@hsr.it
Cameron	Peter	peter.cameron@med.monash.edu.au
Carbayo Lozano	Guillermo	guillermobilbo@gmail.com
Carbonara	Marco	marco.carbonara@gmail.com
Castaño-León	Ana M.	ana.maria.castano.leon@gmail.com
Cavallo	Simona	cavallosimona1@gmail.com
Chevallard	Giorgio	giorgio.chevallard@ospedaleniguarda.it
Chieregato	Arturo	arturo.chieregato@ospedaleniguarda.it
Citerio	Giuseppe	giuseppe.citerio@unimib.it
Clusmann	Hans	hclusmann@ukaachen.de
Coburn	Mark Steven	mark.coburn@ukbonn.de
Coles	Jonathan	jpc44@wbic.cam.ac.uk
Cooper	Jamie D.	jamie.cooper@monash.edu
Correia	Marta	Marta.Correia@mrc-cbu.cam.ac.uk

Čović	Amra	amra.covic@med.uni-goettingen.de
Curry	Nicola	nicola.curry@ouh.nhs.uk
Czeiter	Endre	endre.czeiter@gmail.com
Czosnyka	Marek	mc141@medschl.cam.ac.uk
Dahyot-Fizelier	Claire	c.dahyot-fizelier@chu-poitiers.fr
Dark	Paul	paul.m.dark@manchester.ac.uk
Dawes	Helen	hdawes@brookes.ac.uk
De Keyser	Véronique	veronique.dekeyser@uza.be
Degos	Vincent	vincent.degos@aphp.fr
Della Corte	Francesco	dellacorte.f@gmail.com
den Boogert	Hugo	Hugo.denBoogert@radboudumc.nl
Depreitere	Bart	bart.depreitere@uzleuven.be
Đilvesi	Đula	djuladjilvesi@gmail.com
Dixit	Abhishek	ad825@cam.ac.uk
Donoghue	Emma	emma.donoghue@monash.edu
Dreier	Jens	jens.dreier@charite.de
Dulière	Guy-Loup	glduliere@gmail.com
Ercole	Ari	ae105@cam.ac.uk
Esser	Patrick	pesser@brookes.ac.uk
Ezer	Erzsébet	ezererzsebet@yahoo.com
Fabricius	Martin	fabricius@dadlnet.dk
Feigin	Valery L.	valery.feigin@aut.ac.nz
Foks	Kelly	k.foks@erasmusmc.nl
Frisvold	Shirin	Shirin.Kordasti@unn.no
Furmanov	Alex	alexpuil@yahoo.com
Gagliardo	Pablo	pablog@fivan.org
Galanaud	Damien	galanaud@gmail.com
Gantner	Dashiell	dashiell.gantner@monash.edu
Gao	Guoyi	gao3@sina.com
George	Pradeep	george@incf.org
Ghuysen	Alexandre	A.Ghuysen@chu.ulg.ac.be
Giga	Lelde	lelde.giga@inbox.lv
Glocker	Ben	b.glocker@imperial.ac.uk
Golubović	Jagoš	jagosgolubovic@gmail.com
Gomez	Pedro A.	pagolopez@gmail.com
Gratz	Johannes	johannes.gratz@meduniwien.ac.at
Gravesteijn	Benjamin	b.gravesteijn@erasmusmc.nl
Grossi	Francesca	francesca.grossi@libero.it
Gruen	Russell L.	russell.gruen@anu.edu.au
Gupta	Deepak	drdeepakgupta@gmail.com
Haagsma	Juanita A.	j.haagsma@erasmusmc.nl
Haitsma	Iain	i.haitsma@erasmusmc.nl
Helbok	Raimund	Raimund.Helbok@tirol-kliniken.at

Helseth	Eirik	EHELSETH@ous-hf.no
Horton	Lindsay	lindsay.horton@stir.ac.uk
Huijben	Jilske	j.a.huijben@erasmusmc.nl
Hutchinson	Peter J.	pjah2@cam.ac.uk
Jacobs	Bram	b.jacobs@umcg.nl
Jankowski	Stefan	Stefan.Jankowski@sth.nhs.uk
Jarrett	Mike	mike.jarrett@quesgen.com
Jiang	Ji-yao	jiyaojiang@126.com
Johnson	Faye	faye.johnson@live.co.uk
Jones	Kelly	kejones@aut.ac.nz
Karan	Mladen	mladjokaran@gmail.com
Kolias	Angelos G.	angeloskolias@gmail.com
Kompanje	Erwin	erwinkompanje@me.com
Kondziella	Daniel	Daniel.Kondziella@regionh.dk
Kornaropoulos	Evgenios	ek481@cam.ac.uk
Koskinen	Lars-Owe	Lars-Owe.Koskinen@umu.se
Kovács	Noémi	kovacs.noemi@pte.hu
Lagares	Alfonso	algadoc@yahoo.com
Lanyon	Linda	lindal@incf.org
Laureys	Steven	steven.laureys@ulg.ac.be
Lecky	Fiona	f.e.lecky@sheffield.ac.uk
Ledoux	Didier	dledoux@chu.ulg.ac.be
Lefering	Rolf	Rolf.Lefering@uni-wh.de
Legrand	Valerie	Valerie.Legrand@iconplc.com
Lejeune	Aurelie	aurelie.lejeune@chru-lille.fr
Levi	Leon	llevi@rambam.health.gov.il
Lightfoot	Roger	Roger.Lightfoot@uhs.nhs.uk
Lingsma	Hester	h.lingsma@erasmusmc.nl
Maas	Andrew I.R.	andrew.maas@uza.be
Maegele	Marc	Marc.Maegele@t-online.de
Majdan	Marek	mmajdan@truni.sk
Manara	Alex	Alex.Manara@nbt.nhs.uk
Manley	Geoffrey	ManleyG@ucsf.edu
Maréchal	Hugues	Hugues.Marechal@chrcitadelle.be
Martino	Costanza	costmartino74@gmail.com
Mattern	Julia	Julia.Mattern@med.uni-heidelberg.de
McMahon	Catherine	Catherine.McMahon@thewaltoncentre.nhs.uk
Melegh	Béla	bela.melegh@aok.pte.hu
Menon	David	dkm13@cam.ac.uk
Menovsky	Tomas	tomas.menovsky@uza.be
Mikolic	Ana	a.mikolic@erasmusmc.nl
Misset	Benoit	Benoit.Misset@chuliege.be
Muraleedharan	Visakh	visakh@incf.org

Murray	Lynnette	lynnette.murray@monash.edu
Nair	Nandesh	nandesh.nair@uza.be
Negru	Ancuta	negruancu@gmail.com
Nelson	David	david.nelson@karolinska.se
Newcombe	Virginia	vfjn2@cam.ac.uk
Nieboer	Daan	d.nieboer@erasmusmc.nl
Nyirádi	József	nyiradi.jozsef@pte.hu
Oresic	Matej	matej.oresic@oru.se
Ortolano	Fabrizio	lupeda@gmail.com
Otesile	Olubukola	o.otesile@sheffield.ac.uk
Palotie	Aarno	aarno.palotie@helsinki.fi
Parizel	Paul M.	paul.parizel@uantwerpen.be
Payen	Jean-François	Jean-Francois.Payen@ujf-grenoble.fr
Perera	Natascha	perera@arttic.eu
Perlbarg	Vincent	vincent.perlbarg@gmail.com
Persona	Paolo	ppersona75@gmail.com
Peul	Wilco	W.C.Peul@lumc.nl
Piippo-Karjalainen	Anna	anna.piippo@hus.fi
Pirinen	Matti	matti.pirinen@helsinki.fi
Ples	Horia	horia.ples@neuromed.ro
Polinder	Suzanne	s.polinder@erasmusmc.nl
Pomposo	Inigo	inigo.pomposo@osakidetza.net
Posti	Jussi P.	jussi.posti@tyks.fi
Puybasset	Louis	louis.puybasset@aphp.fr
Rădoi	Andreea	aradoi@neurotrauma.net
Ragauskas	Arminas	telematics@ktu.lt
Raj	Rahul	rahul.raj@hus.fi
Rambadagalla	Malinka	malinka.rambadagalla@gmail.com
Rehorčíková	Veronika	rehorcikova@gmail.com
Rhodes	Jonathan	jrhodes1@staffmail.ed.ac.uk
Richardson	Sylvia	sylvia.richardson@mrc-bsu.cam.ac.uk
Richter	Sophie	sr773@cam.ac.uk
Ripatti	Samuli	samuli.ripatti@helsinki.fi
Rocka	Saulius	saulius.rocka@mf.vu.lt
Roe	Cecilie	e.c.t.roe@medisin.uio.no
Roise	Olav	olav.roise@medisin.uio.no
Rosand	Jonathan	jrosand@partners.org
Rosenfeld	Jeffrey	J.Rosenfeld@alfred.org.au
Rosenlund	Christina	chrisstenrose@gmail.com
Rosenthal	Guy	rosenthalg@hadassah.org.il
Rossaint	Rolf	RRossaint@ukaachen.de
Rossi	Sandra	sandrarossi0@gmail.com
Rueckert	Daniel	d.rueckert@imperial.ac.uk

Rusnák	Martin	mrusnak@igeh.org
Sahuquillo	Juan	sahuquillo@neurotrauma.net
Sakowitz	Oliver	oliver.sakowitz@gmail.com
Sanchez-Porras	Renan	renan_md@hotmail.com
Sandor	Janos	sandor.janos@sph.unideb.hu
Schäfer	Nadine	Nadine.Schaefer@uni-wh.de
Schmidt	Silke	silke.schmidt@uni-greifswald.de
Schoechl	Herbert	Herbert.Schoechl@auva.at
Schoonman	Guus	g.schoonman@tsz.nl
Schou	Rico Frederik	rico@mymedic.dk
Schwendenwein	Elisabeth	elisabeth.schwendenwein@meduniwien.ac.at
Sewalt	Charlie	c.sewalt@erasmusmc.nl
Skandsen	Toril	toril.skandsen@ntnu.no
Smielewski	Peter	ps10011@cam.ac.uk
Sorinola	Abayomi	sorinola_abayomi@hotmail.com
Stamatakis	Emmanuel	eas46@cam.ac.uk
Stanworth	Simon	simon.stanworth@nhsbt.nhs.uk
Kowark	Ana	akowark@ukaachen.de
Stevens	Robert	rstevens@jhmi.edu
Stewart	William	william.stewart@glasgow.ac.uk
Steyerberg	Ewout W.	e.steyerberg@erasmusmc.nl
Stocchetti	Nino	stocchet@policlinico.mi.it
Sundström	Nina	Nina.Sundstrom@vll.se
Takala	Riikka	riikka.takala@tyks.fi
Tamás	Viktória	tamas.viktoria@pte.hu
Tamosuitis	Tomas	tomas.tamosuitis@kaunoklinikos.lt
Taylor	Mark Steven	marktrnava@gmail.com
Te Ao	Braden	braden.teao@aut.ac.nz
Tenovuo	Olli	olli.tenovuo@tyks.fi
Theadom	Alice	alice.theadom@aut.ac.nz
Thomas	Matt	Matt.Thomas@nbt.nhs.uk
Tibboel	Dick	d.tibboel@erasmusmc.nl
Timmers	Marjolijn	mtimmers@hotmail.com
Tolias	Christos	christos.tolias@nhs.net
Trapani	Tony	tony.trapani@monash.edu
Tudora	Cristina Maria	cristina.tudora@neuromed.ro
Unterberg	Andreas	Andreas.Unterberg@med.uni-heidelberg.de
Vajkoczy	Peter	Peter.Vajkoczy@charite.de
Valeinis	Egils	Egils.Valeinis@latnet.lv
Vallance	Shirley	S.Vallance@alfred.org.au
Vámos	Zoltán	azozoka@gmail.com
Van der Jagt	Mathieu	m.vanderjagt@erasmusmc.nl
van der Naalt	Joukje	j.van.der.naalt@umcg.nl

Van der Steen	Gregory	gregory@webstone.be
van Dijck	Jeroen T.J.M.	j.van.dijck@haaglandenmc.nl
van Essen	Thomas A.	T.A.van_Essen@lumc.nl
Van Hecke	Wim	wim.vanhecke@icometrix.com
van Heugten	Caroline	Caroline.vanheugten@maastrichtuniversity.nl
Van Praag	Dominique	dominique.vanpraag@uza.be
van Wijk	Roel	roel-van-wijk@ziggo.nl
Vande Vyvere	Thijs	thijs.vandevyvere@icometrix.com
Vargiolu	Alessia	neurorianimazione@hsgerardo.org
Vega	Emmanuel	emmanuel.vega@chru-lille.fr
Velt	Kimberley	k.velt@erasmusmc.nl
Verheyden	Jan	jan.verheyden@icometrix.com
Vespa	Paul M.	PVespa@mednet.ucla.edu
Vik	Anne	anne.vik@ntnu.no
Vilcinis	Rimantas	rimantas.vilcinis@kaunoklinikos.lt
Volovici	Victor	v.volovici@erasmusmc.nl
von Steinbüchel	Nicole	nvsteinbuechel@med.uni-goettingen.de
Voormolen	Daphne	d.voormolen@erasmusmc.nl
Vulekovic	Petar	pvulekovic@gmail.com
Wang	Kevin K.W.	kawangwang17@gmail.com
Wiegers	Eveline	e.wiegers@erasmusmc.nl
Williams	Guy	gbw1000@wbic.cam.ac.uk
Wilson	Lindsay	l.wilson@stir.ac.uk
Winzeck	Stefan	sw742@cam.ac.uk
Wolf	Stefan	stefan.wolf@charite.de
Yang	Zhihui	zhihuiyang@ufl.edu
Ylén	Peter	peter.ylen@vtt.fi
Younsi	Alexander	alexander.younsi@med.uni-heidelberg.de
Zeiler	Frederick A.	umzeiler@myumanitoba.ca
Ziverte	Agate	agate.ziverte@inbox.lv
Zoerle	Tommaso	tommaso.zoerle@policlinico.mi.it
